# Supplementary figures and images for: Some t-tests for N-of-1 trials with serial correlation
Source: PLoS One. 2020 Feb 4;15(2):e0228077. doi: 10.1371/journal.pone.0228077 (PMC6999905; doi:10.1371/journal.pone.0228077)

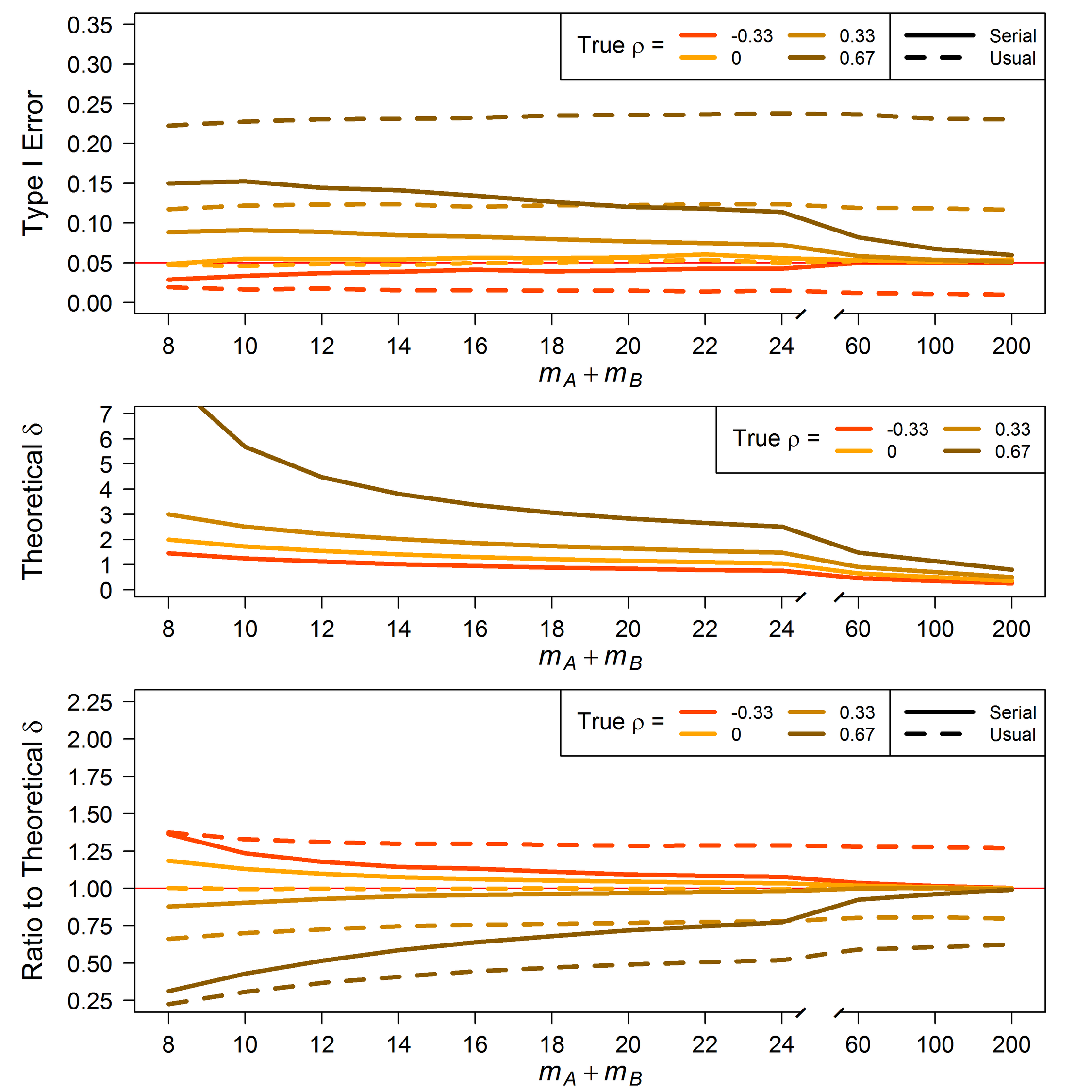

Supplement: S1 Fig — Type I error (top) and theoretical effect size, δ, computed using Eq 9 for 80% power with a one-sided 5% significance level test for a given m; note, mA = mB (middle). Ratios (bottom) of serial and usual δ (estimated from simulation) to the theoretical δ under same conditions as the theoretical δ. (TIF) [file pone.0228077.s001.tif]

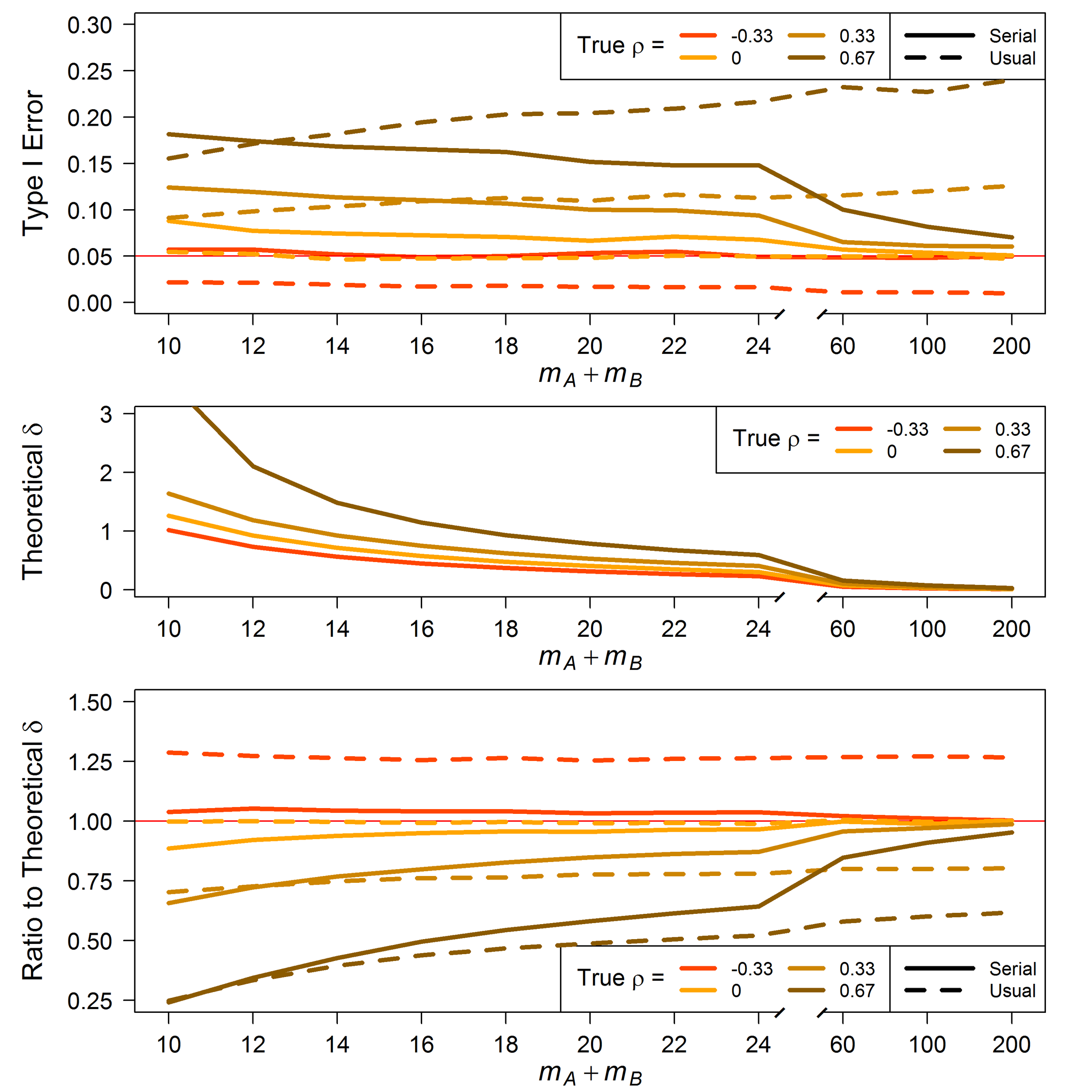

Supplement: S2 Fig — Type I error (top) and theoretical effect size, δ, computed using Eq 15 for 80% power with a one-sided 5% significance level test for a given m; note, mA = mB (middle). Ratios (bottom) of serial and usual δ (estimated from simulation) to the theoretical δ under same conditions as the theoretical δ. (TIF) [file pone.0228077.s002.tif]
