# Supplementary material for: Some t-tests for N-of-1 trials with serial correlation
Source: PLoS One. 2020 Feb 4;15(2):e0228077. doi: 10.1371/journal.pone.0228077 (PMC6999905; doi:10.1371/journal.pone.0228077)
Supplement: Attachment — Submitted filename: PONE-D-19-28132 Response to Reviewers.docx [file pone.0228077.s003.docx]

2. We noted in your submission details that a portion of your manuscript may have been presented or published elsewhere. [Table 4 reproduces a portion of Table 1 from Jaeschke et al. (1991). We re-analyze their data with the proposed statistical methods.]

Please clarify whether this  publication was peer-reviewed and formally published. If this work was previously peer-reviewed and published, in the cover letter please provide the reason that this work does not constitute dual publication and should be included in the current manuscript.

This work was peer-reviewed and formally published. In the manuscript we provide a reference. This does not constitute a dual publication. We excerpt data from their Table 1, and re-analyze the data with our proposed method.

Additional Editor Comments (if provided):

Even though the manuscript is well-written and statistically sound it is unclear what the overall impact of this approach would be in terms of real-world applications. If you could provide an additional real-world example that would satisfy review #1 concerns that would be appreciated.

To better illustrate impact of this approach on real-world applications, we added additional information in the fibromyalgia example, and added a paragraph to the *Implications* subsection in the Discussion.

Regarding adding an additional example: We are not opposed to an adding another real-world example. Indeed, we have one study that presents 7 N-of-1 trials (i.e., 7 patients). With some work and the addition of a co-author, this example can be included. We think, though, that the structure and size of this additional example is very similar to our fibromyalgia example. (The data were originally published in *The Annals of Pharmacotherapy* in 2005 by Pereira et al. DOI 10.1345/aph.1G003 ). Thus, Reviewer #1 may still have the same critique of the additional example. Please allow us to defend, as sufficient, the 2 examples we originally included. Below, we provide text (in black) that speaks to the rationale for including these examples. Our response to you and Reviewer #1 is in green.

--- Regarding the fibromyalgia example ---

The authors individually applied a one-sided (usual) paired *t*-test to determine benefit of amitriptyline for each patient. We re-analyzed each patient’s data… {Reviewer #1 writes, “… many subjects are involved… .” Each patient was analyzed individually in the original report by Jaeschke *et al.* (1991).}

However, since this serial *t*-test requires at least 4 pairs of treatments, we could use only 6 of the original 23 patients. {Reviewer #1 writes, “…the methods you develop are for single case n-of-1 trials with many treatment periods per subject.” The *t*-tests we develop are also for *N*-of-1 trials with few observations. We state the lower limit of observations at the bottom of each *t*-statistic. This fibromyalgia example shows how inferences are sharpened when accounting for serial correlation in small-sized *N*-of-1 trials.}

--- Regarding the discounting example ---

The discounting example shows how *each* of the four serial *t*-tests can be applied. The original article (Landes et al., 2012) analyzed each patient independently. We also apply the serial *t*-tests to each patient, independent of the others. We show what each test looks like with one (real) example dataset in Table 5. We summarize the individual results of all the patients in Table 6, rather than reporting the results of an aggregated analysis. Again, we think this example deals with a small-sized *N*-of-1 situation in which an individual’s data is analyzed by itself.

1. Is the manuscript technically sound, and do the data support the conclusions?

The manuscript must describe a technically sound piece of scientific research with data that supports the conclusions. Experiments must have been conducted rigorously, with appropriate controls, replication, and sample sizes. The conclusions must be drawn appropriately based on the data presented.

Reviewer #1: Partly We are unsure how to address this critique. Hopefully Reviewer #1 provided more details in Review Comments.

Reviewer #2: Yes

2. Has the statistical analysis been performed appropriately and rigorously?

Reviewer #1: Yes

Reviewer #2: Yes

3. Have the authors made all data underlying the findings in their manuscript fully available?

The [PLOS Data policy](https://urldefense.proofpoint.com/v2/url?u=http-3A__www.plosone.org_static_policies.action-23sharing&d=DwMGaQ&c=27AKQ-AFTMvLXtgZ7shZqsfSXu-Fwzpqk4BoASshREk&r=m5mAynaTMM-dGh2Ve2XBcWmqoBMmcR2ey49uweMsugM&m=YJpgihPfa8eX2BIPNSWHPgF6JdpJd36xLbcj4fNiJJU&s=J_QOdGkbhgaFfG4IJ1zsXVZbteOUE_TI8J7v7olYAP4&e=) requires authors to make all data underlying the findings described in their manuscript fully available without restriction, with rare exception (please refer to the Data Availability Statement in the manuscript PDF file). The data should be provided as part of the manuscript or its supporting information, or deposited to a public repository. For example, in addition to summary statistics, the data points behind means, medians and variance measures should be available. If there are restrictions on publicly sharing data—e.g. participant privacy or use of data from a third party—those must be specified.

Reviewer #1: Yes

Reviewer #2: Yes

4. Is the manuscript presented in an intelligible fashion and written in standard English?

PLOS ONE does not copyedit accepted manuscripts, so the language in submitted articles must be clear, correct, and unambiguous. Any typographical or grammatical errors should be corrected at revision, so please note any specific errors here.

Reviewer #1: Yes

Reviewer #2: Yes

5. Review Comments to the Author

Reviewer #1

Major points

In my opinion, practical aspects of what you are proposing should be discussed more fully.

1) For example, as far as I can see, the methods you develop are for single case n-of-1 trials with many treatment periods per subject. Yet the practical example of fibromyalgia that you use to illustrate your approach fulfills neither of these conditions: many subjects are involved with and most if not all subject have short series for the type of approach your are developing. Of course, one advantage of developing methods for single subjects is that they can be used from the beginning. Methods for many subjects may have to wait a long time for subjects to accrue during which time early patients may be awaiting a decision.

We are a little confused by this comment: “…are for single case n-of-1 trials with *many treatment periods* per subject” (emphasis ours). We focused on smaller sized trials – those having a dozen or less observations per treatment (see the 4^th^ paragraph in the Introduction). We also state the minimum number of observations for each of the 4 serial *t*-tests after defining the degrees of freedom. The 6 fibromyalgia patients in our Table 4 each provided between 4 to 6 observations for analysis. The original article (Jaeschki *et al.*, 1991) individually analyzed each patient. We also individually analyzed the data from each of these patients. Then we point to an aggregate *N*-of-1 method that used the same data (Zucker *et al.*, 1997) to illustrate how the serial *t*-tests can sharpen inference over an aggregate *N*-of-1 method that does not account for serial correlation.

In the fibromyalgia example (Section 4.1), we added some language to better highlight that we are using the serial *t*-tests on an individual-by-individual basis, and that the method employed by Zucker *et al.* (1997) is an aggregate *N*-of-1 method; whereas ours is not.

Finally, in the *Implications* subsection in the Discussion, we added a paragraph that describes the practical impact of using these serial *t*-tests when also considering another method that does not account for serial correlation.

2) Nevertheless, even if you specifically wish to avoid using information from other patients when making a decision for a given one, I think at the very least you ought to consider whether information about nuisance parameters (for example correlation coefficients) at least could be shared between patients.

Agreed, and thank you. We now include a Discussion paragraph describing how to overcome the bias in $\rho$ when $m$ is small.

3) If the design in pairs is used and treatment order is randomised, I would have thought that the naive standard errors for a t-test would be calculated approximately correctly. This is generally what is assumed in experiments subject to temporal or spatial (as in agriculture) correlation. Are you able to check this? Of course, the serial correlation may be more of a problem for a short single series than for the case of multiple sequences considered in the paper by Senn that you cited.

We are able to check this. In the figure below, we show observed and predicted standard errors of a mean difference for *N*-of-1 trials like those illustrated in the top panels of our Figure 1. Points represent standard deviations of mean differences; i.e., observed standard errors. Each point is based on 1000 mean differences (i.e., 1000 simulated *N*-of-1 trials). Lines represent standard errors predicted by formulas. The *N*-of-1 trials, each with *m* cross-overs, have either no serial correlation (squares) or a serial correlation of 0.33 (circles). The black line is the predicted naïve standard error from Eqn 8 in Senn (2017): ${SE}_{naive}=\sqrt{{2\sigma^{2}}/k}$ (where $k$ is our $m$.) The green lines are from the denominator of Eqn 8 in our manuscript: $\sqrt{{c_{L}(\rho)\times s^{2}}/{b_{L}(\rho)}}$ , replacing $s$ with $\sigma$. Increasing *m* does not improve the downward bias in the naïve standard error estimator. Here, the solid green line is about 1.4 times the black line across *m*. We are sending the R code that produced this mini-simulation; the file name is “PONE-D-19-28132 Response to Reviewer 1 - mini-simulation.R”.

Minor points

Articles to go with nouns are missing in many places. Some examples are

1) L281 The variance
2)L361 The order
3) L496 The variance

We have tried to add articles throughout.

Thank you for your review. Because of your expertise in this area, we are particularly glad we had you review it.

Reviewer #2

Reviewer #2: Thank you for this contribution.
I propose a few minor changes that should improve the robustness of this manuscript:

Abstract: You do well to highlight the need for this type of work (indeed within patient correlation has been a concern), but the statement "existing methods require simulation..." contradicts your analysis, which itself involves MC simulations. I would clarify your intentions here either in the manuscript or consider revising this portion of the abstract.

We use MC simulation to evaluate statistical properties of the proposed *t*-tests. The *t*-tests are still formulae, and do not need simulation computation to get a result for a single patient. The “existing methods” to which we refer *do* require simulation to get a result for a single patient. We have adjusted the abstract in hopes of better clarify the distinction.

Original: We then evaluate and compare their Type I and II errors and interval estimators to those of usual *t*-tests analogues via Monte Carlo simulation.

Updated: We then use Monte Carlo simulation to evaluate the statistical properties of these serial *t*-tests, namely, Type I and II errors, and confidence interval widths, and compare the properties to those from analogous usual *t*-test.

Intro: Broaden your review of how many observations within patients are normal- I know some of my collaborators have seen upwards of 30. Review Blackston et al. and Nikles' work for how aggregated nof1s perform regardless of sample size and consider citing.

Looking back at the Gabler et al. (2011) review, we see they provide information on the number of observations taken on an individual. For both Gabler et al. and Punja et al (2016), we incorrectly assumed the number of observations were 2× the number of crossovers or cycles. We have now corrected a portion of the Introduction and Discussion.

Thank you for bring the Blackston et al. and Nikles et al. articles to our attention. After going through them both, we did not find where they spoke of how many observations are typically taken on an individual.

Nikles et al. (2011) advocate aggregating *N*-of-1trials. No doubt, aggregating *N*-of-1 trials can reduce Type I and II errors for individual level inference (given the data reasonably satisfy the underlying assumptions of the statistical model). However, our work focuses on a method that can be used on a *single* *N*-of-1 trial. With respect to the number of observations per individual, Nikles and company note in their Table 1 that the number of cycles (i.e,. crossovers) individual experiences should be (at least?) 3. They do not indicate how many observations should be taken in each cycle.

Blackston et al. (2019) also considers aggregated *N*-of-1 trials, and considers 3 cycles in their simulations. The 3 cycles correspond to 6 observations per individual, summarized to 3 differences. Their simulation scenario is like our Fig. 1a, noting our “block” is the same as a cycle. Their scenario assumes one observation is produced from a treatment in a cycle. From the *N*-of-1 trials with multiple crossovers that we have seen, Blackston & company’s simulation has realistic assumptions on trial design. All this to say that small sized *N*-of-1 trials seem to be the rule rather than exception.

Methods: Good work overall. Please review Huber et al's work in 2007 on amitriptyline in an Nof1 setting and cite if necessary.

Thank you!

We reviewed Huber et al. (2007). Each *N*-of-1 trial in their study provided 3 differences between the placebo and amitriptyline (i.e., “3 paired crossovers (6 treatment periods)” pg 1127). Our method needs at least 4 observations since estimating the serial correlation requires an extra observation. Additionally, Huber included data from all *N*-of-1 patients in their analysis (i.e., an aggregated *N*-of-1 method), rather than individually analyzing each patient. For these two reasons, we do not feel it will add to the paper to include this paper. From the amitriptyline/fibromyalgia example in our paper, Jaeschke et al. (1991) analyzed each patient individually. Further, 6 of the original 23 patients provided at least 4 observations, allowing us to use the serial *t*-tests.

Thanks.

And we thank you. You brought some more literature to our attention, and helped us clear up some things.
